# Parasitological, Serological and Molecular Study of *Dirofilaria immitis* in Domestic Dogs, Southeastern Iran

**Published:** 2017

**Authors:** Mehdi BAMOROVAT, Iraj SHARIFI, Majid FASIHI HARANDI, Saeed NASIBI, Balal SADEGHI, Javad KHEDRI, Mohammad Ali MOHAMMADI

**Affiliations:** 1. Leishmaniasis Research Center, Kerman University of Medical Sciences, Kerman, Iran; 2. Research Center for Hydatid Disease in Iran, Kerman University of Medical Sciences, Kerman, Iran; 3. Dept. of Food Hygiene and Public Health, Faculty of Veterinary Medicine, Shahid Bahonar University of Kerman, Kerman, Iran; 4. Dept. of Pathobiology, Faculty of Veterinary Medicine, Shahid Bahonar University of Kerman, Kerman, Iran

**Keywords:** *Dirofilaria immitis*, Domestic dog, Diagnostic tests, Iran

## Abstract

**Background:**

Dirofilariasis is a serious and potentially deadly condition in dogs and one of the zoonotic filarial infections, which inadvertently affects the humans. The objectives of this study were to determine the seroprevalence and the molecular identity of dirofilariasis in Kerman Province, southeastern Iran between Jul and Aug 2013.

**Methods:**

A hundred and forty-nine domestic dogs were randomly selected and five ml blood samples were taken from each dog. One ml of anticoagulant (EDTA) was used for each test in the parasitological study (modified Knott’s test) and sera samples were examined, using ELISA kit to detect *Dirofilaria immitis* antigen. Extracted DNA of all positive blood samples was used for molecular characterization and sequencing.

**Results:**

Four (2.7%) domestic dogs of the total 149 domestic dogs were infected with micofilariae of *D. immitis*, while the serological study showed 8 (5.4%) domestic dogs were infected with *D. immitis*. No significant difference, however, was found between dirofilariasis infection and gender. On the other hand, a significant difference was observed between dirofilariasis infection and age *(P*<0.05*).* Based on the PCR findings, among the total specimens, 6 positive samples were characterized as *D. immitis.*

**Conclusion:**

Dirofilariasis occurred when there was low endemicity in the dogs. Such dogs could be a potential source of infection for humans. These findings could help in better understanding of the epidemiological aspects of *D. immitis* in the southeastern parts of Iran.

## Introduction

Dirofilariasis is a zoonotic infection caused by the filarial nematodes from genus *Dirofilaria*, which includes more than 40 different species. Dirofilariasis can be an infectious zoonotic disease when humans are bitten by culicid mosquitoes harboring infective third-stage larvae (microfilariae), due to *D. immitis.* Infected humans have been reported essentially in areas that have high prevalent dirofilariasis (1, 2) and particularly in tropical, subtropical and to some extent in temperate regions, throughout the world (3). Although infections in humans often undergo truncated development, these infections are sometimes associated with pathogenic manifestation and are often severe (2). This disease has also been reported as an emerging zoonosis in many countries worldwide such as Iran (3–5). In various parts of Iran, *D. immitis* is reported in dogs, humans, and cats with different rates (3,6, 7). In addition to many contributing factors that cause this disease, *Dirofilaria* species have tendency to spread in various environmental conditions such as climate changes and availability of vector species. The increasing temperature caused by climate changes can affect the prevalence and spread of mosquitoes and therefore the transmission of this (8,9).

There are several methods for the diagnosis of dirofilariasis in dogs. Blood smears or concentration methods such as Knott’s technique or the filtration test are applicable for all species with blood-circulating microfilariae that include morphological identification. ELISA and immunochromatographic tests that can detect circulating antigens in adult female nematodes are currently only available for *D. immitis*. In the last decade, several techniques have been developed for specific diagnosis of various species of filarioids in terms of molecular identifications (10). Currently, numerous enzyme-linked immunosorbent assay kits are available to diagnose heartworms in dogs. However, molecular techniques, in particular, PCR-based methods, provide an alternative detection possibility which are precise and sensitive for identification purposes (11). Nowadays, definitive identification of *D. immitis* is possible for canine and feline with the help of genetic techniques (12).

This study was performed to investigate the prevalence rate of *D. immitis* infection in domestic dogs by a modified Knott’s test and antigen-detecting ELISA along with molecular identity of dirofilariasis in Kerman, southeastern Iran.

## Materials and Methods

### Ethical statement

This project (no. 91/56) was reviewed and approved by the Ethical Committees of the Kerman University of Medical Sciences and Leishmaniasis Research Center (Kerman, Iran). The owners of the dogs participating in the survey were informed of the research goals and signed the consent form before sampling.

### Study design

The cross-sectional study was undertaken systematically between Jul and Aug 2013, on domestic dogs in Kerman, southeastern Iran. This largest Iran’s Province consisting an area of 180.726 km^2^, hot semi-arid climate and 2.9 million populations, located in the southeastern part of Iran. Overall, 149 domestic dogs were randomly selected and blood samples were taken from each dog. Then, a questionnaire was completed for each of them recording their sex, age and clinical signs of dirofilariasis. The age of the dogs was determined by examining dental formula (13). Most clinical presentations were related to respiratory and cardiac alterations as declared by the dogs’ owner. Five ml blood samples were taken from the cephalic vein of each and transported to the Department of Medical Parasitology at School of Medicine at Kerman University of Medical Sciences.

### Parasitological study

One ml of anticoagulant EDTA was used for the parasitological study. Blood samples of domestic dogs were examined using a modified Knott’s technique. In Knott’s method, 1 ml of blood was mixed with 9 ml of 2% formaldehyde and then was thoroughly shaken for RBC hemolysis. After centrifugation, Giemsa stain was added and the mixture was examined to detect any possible microfilariae, under a light microscope (14).

### Serological examination

For each dog, four ml blood sample was used for serological examination. Blood samples were centrifuged at 3000 rpm for 3–5 min and the separated sera were stored at −20 °C for subsequent serological tests. Sera were examined using an ELISA kit (DiroCHEK, Pfizer, USA) for detecting adult *D. immitis* antigen following the manufacturer’s instructions. The kit was designed to detect the circulating antigen in adult female worms. DiroCHEK will not give false positive results due to the presence of other parasites, drugs, supplements or vaccines.

### Molecular study

#### DNA purification and polymerase chain reaction

Genomic DNA of all serological positive blood samples was extracted, using a DNA extraction kit (Accu-Prep, Bioneer, Korea) following the manufacturer’s instructions. The CO1 sequences were generated using the primer pair DirF 5′-CCTTTGAGTGTAGAGGGTCAGC-3′ and DirR 5′-ATTCCGCTCAAACCTCCAAT-3′. Each PCR reaction was carried out with the master mix in 25 μl of total reaction. Based on the following conditions: the initial denaturation at 94 °C for 3 min, followed by 40 cycles including denaturation at 94 °C for 30 sec, annealing at 58 °C for 35 sec, extension at 72 °C for 1 min, and the final extension at 72 °C for 5 min. At the end, 5 μl of the reaction mix was analyzed by 1.5% agarose gel electrophoresis to confirm PCR-specific amplification.

### Sequencing and phylogenetic analysis

PCR products were sent to Macrogen Company (Korea) to be purified and sequenced with a Big Dye Kit (ABI) using the PCR primers. Sequence similarity searching for each single sample was performed using the NCBI BLAST program. Analyses of multiple sequence alignments were done using the programs BioEdit ver.6 (15) and MEGA ver.6 (16). For distance analysis, the Kimura-2 parameter model was used to construct the distance matrix and the tree was inferred using the Maximum Likelihood approach. Bootstrap resampling 1000 was performed and a bootstrap consensus tree was produced.

### Statistical analysis

Data analysis was performed using SPSS software ver.21 (Chicago, IL, USA) and positive samples were set as an outcome variable. Sex and age were used as independent variables. A primary screening was performed using two K contingency tables (cross-tab) of exposure variables by chi-square and fisher exact tests and *P<0.05* was defined significant.

## Results

### Parasitological survey

Four domestic dogs (2.7%) showed microfilariae of *D. immitis* when the modified Knott’s test was performed.

### Seroepidemiological survey

The results showed that 8 (5.4%) of the total 149 domestic dogs were infected with *D. immitis*. Out of the 149 examined dogs, 57 (38.3%) were male and 92 (61.7%) females classified into four age groups (< 1 yr, 1–3 yr, >3–5 yr and >5 yr) (Table 1). No significant difference was found between dirofilariasis infection and gender. On the other hand, there was a significant difference between dirofilariasis and age (*P*<*0.05*).

**Table 1:** The seroprevalence of dirofilariasis caused by *D. immitis* in domestic dogs in the city and suburbs of Kerman, Kerman Province, southeast of Iran, 2013

**Characteristics**		**Positive No. (%)**	**Negative No. (%)**	**Total No. (%)**
Age (yr)	<1	0 (0)	39 (100)	**39 (26.2 )**
	1–3	0 (0)	48 (100)	**48 (32.2 )**
	3–5	5 (11.9)	37 (88.1)	**42 (28.2)**
	>5	3 (15)	17 (85)	**20 (13.4)**
Sex	Male	3 (5.3)	54 (94.7)	**57 (38.3)**
	Female	5 (5.4)	87 (94.6)	**92 (61.7)**
Total		8 (5.4)	141(94.6)	**149 (100)**

### PCR, Sequencing and Phylogenetic Analysis

**Fig. 1:**
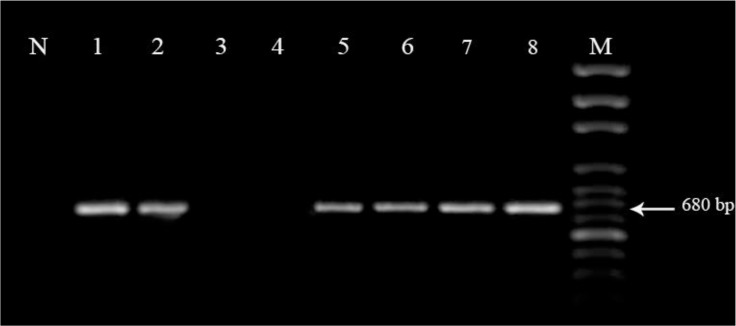
Amplification of partial cytochrome c oxidase subunit 1 in mitochondrial DNA, using primers new DirF and DirR Primers. Six positive isolates that were amplified 680 bp PCR products showed as lanes 1, 2 and 5 to 8, negative isolates (lanes 3 and 4), non-template control (lane N), molecular size marker (lane M)

**Fig. 2:**
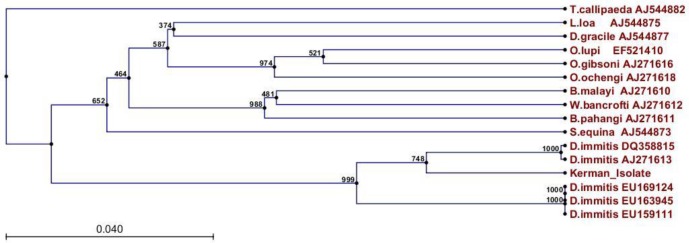
A phylogenetic tree rooted by out-group (*Thelazia callipaeda*), inferred from partial CO1 nucleotide sequences using the Neighbor-Joining method after Kimura2 correction. Scale bar indicates the proportion of sites changing along each branch

Based on PCR findings, among total specimens, six dogs were found positive as *D. immitis*. The aligned portion of the CO1 region of the samples was about 680 bp (Fig. 1). CO1 sequences were submitted to the GenBank under the accession numbers KR870344. Phylogenetic tree rooted by out-group (*Thelazia callipaeda*), inferred from partial cytochrome c oxidase sequences. All *D. immitis* records appeared as sister clade and Kerman filarial isolate close to DQ358815 and AJ271613 records from Italy (Fig. 2).

## Discussion

This study is the first comprehensive assessment of heartworm disease applying combination of methods in dogs in the southeastern Iran. *D. immitis* affects dogs, wild canines, felines and human population in tropical, subtropical and temperate areas throughout the world and creates a potential risk of contracting dirofilariasis in endemic areas (4–6,10,17). A comparison of the historical epidemiological data over the past years shows that significant changes in the prevalence of dirofilariasis have been occurring globally. These variations could be linked to climate change and population dispersion of *Dirofilaria* species vectors*.* In some regions and in existing testing methodologies for diagnosis (18, 19). Besides, microfilariae may flood into the peripheral blood circulation at a certain time of the day or night and hide from the peripheral circulation at other times (14). Since the microfilariae had a nocturnal periodicity, our samples were taken during the night, although this nocturnal periodicity is different in several countries (20). Most infected dogs with *D. immitis* do not show clinical signs of disease, whereas a high proportion demonstrated loss of weight, listlessness and decreased appetite.

Based on the serological finding, the overall prevalence of dirofilariasis was estimated at 5.4%. Although this data presented a lower prevalence compared to other areas of Iran but such prevalence rate is high for this area due to its hot and semi-arid climatic conditions. In this study, no statistically significant difference was observed between dirofilariasis and sex. Moreover, no significant differences by sex have been reported in other studies (21–24). In contrast, some authors (25–28) have reported a significantly higher prevalence in male dogs. Moreover, there was a significant difference between dirofilariasis infection and age (*P*<0.05). Age is an important risk factor for the contraction of the infection. The infection risks for dogs are probably related to the increasing length of the period of their exposure to mosquitoes and the amount of time they spend outdoors (23, 26). Hence, dogs of older age groups display higher infection rate than the younger ones. This finding is consistent with other studies (28–30). We found the highest prevalence in animals > 5 yr old (15%) and the lowest in animals under 1-year-old (0%).

By the parasitological method (modified Knott), four domestic dogs (2.7%) showed microfilariae of *D. immitis* in low levels whereas this figure was higher when the serological method was applied. The differential diagnosis between microfilariae of *D. immitis* was performed regarding the morphological criteria (14). According to previous studies, two species of the genus *Dirofilaria* consisting of *D. immitis* (canine heartworm) and *D. repens* were found in several areas of Iran (6,31, 32). Rarzmaraii et al. characterized canine microfilariae species in East-Azerbaijan Province of Iran based on internal transcribed spacer 2 (33), although Azari-Hamidian et al. described CO1 sequences for third-stage larvae of *D. immitis* from Iranian specimens (31). We did not find any sequence submission on CO1 region in NCBI database to compare phylogeny of Iranian *Dirofilaria* isolates. However, in the present study, all positive samples belonged to *D. immitis* bearing the same sequence. Distance analyses have shown the same phylogeny with other filarial nematode (34, 35). Because of differences in sensitivity and specificity of diagnostic serology and molecular techniques, the possibility of differences in the results of these techniques is inevitable (36).

The transmission rate of dirofilariasis due to various species depends on the presence of infected dogs as the main reservoir host and the availability of principal vectors for transmission of the disease. Therefore, dirofilariasis transmission is influenced by two factors that affect both of the two components of the worms’ life cycle: first, the human behavior with respect to pets and second, climatic factors that allow the presence of competent vector populations and *Dirofilaria* larval developments in these vectors (18, 29). In terms of epidemiology, dirofilariasis is thought-out an emerging zoonotic disease. In humans similar conditions of disease as for dogs, comprising pulmonary and heart diseases have been reported (29).

Rapid and significant changes in the distribution of canine reservoirs have globally been reported and this, in turn, contributes to the expansion of infection to other hosts including humans and feline. Global warming influences the stages of the parasite life cycle in vectors. Therefore, these potential factors along with lack of pet management measures could increase the infection rate in humans. These findings could help in better understanding of epidemiological aspects of *D. immitis* in Kerman, southeastern Iran.

## Conclusion

Despite the dry, hot and semi-arid climatic conditions, the prevalence rate of dirofilariasis is high for this area. Our findings could optimally contribute to a better understanding of the epidemiological aspects of *D. immitis* in this region. This kind of information is essential for planning effective future control program.
